# Distinct Physical Properties of β-Hematin in Two Synthetic Media: Compelling Evidence

**DOI:** 10.1021/acsomega.4c06694

**Published:** 2025-03-19

**Authors:** Julieth Herrera, Karen Edilma García, Valentina Perez, José Francisco Marco, César Barrero Meneses

**Affiliations:** † Institute of Chemistry, Faculty of Natural and Exact Sciences, University of Antioquia, Street 70 # 52−72, Medellín 050010, Colombia; ‡ Institute of Physics, Faculty of Natural and Exact Sciences, University of Antioquia, Street 70 # 52−72, Medellín 050010, Colombia; § Instituto de Química Física Blas Cabrera, CSIC, Serrano 119, Madrid 28006, Spain

## Abstract

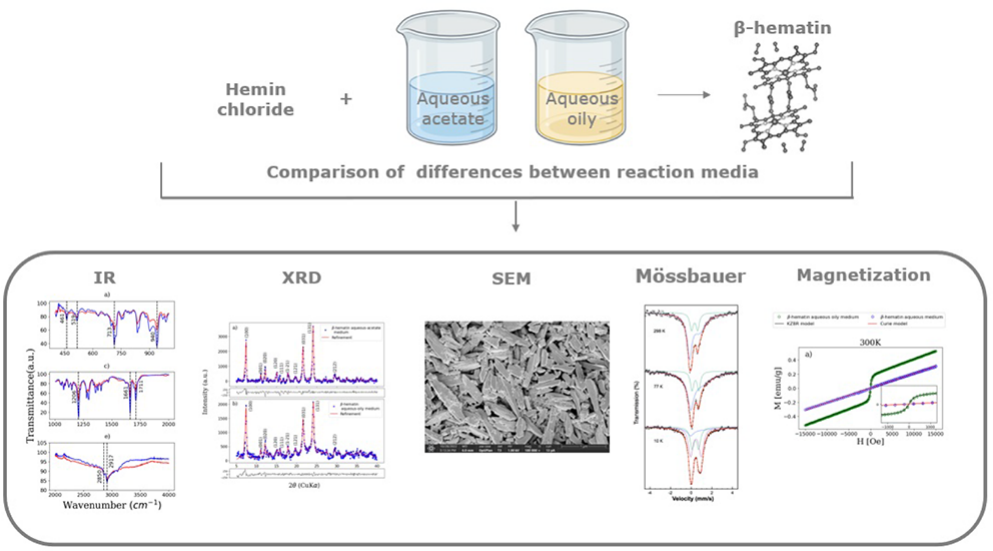

It is now widely accepted that detailed knowledge of the physicochemical characteristics of the β-hematin crystals, i.e., the synthetic versions of the natural hemozoin crystals, is important for understanding their formation, the design of antimalarial medicines, and malarial diagnosis. We report that the overall physical properties exhibited by β-hematins greatly depend on the synthetic media. Here, we synthesize β-hematin from hemin in aqueous-acetate and in aqueous-oily media and characterize their properties by several techniques. Infrared spectra clearly demonstrate the formation of β-hematin in both media. The β-hematin crystals prepared in aqueous-acetate are composed by needle-like particles with average lengths around 760–770 nm; their lattice parameters and unit cell volumes are larger than those reported in the literature. They are paramagnetic at 300 K and antiferromagnetic at very low temperatures. Their Mössbauer spectra at 298 K, 77 K, and 10 K are consistent with the presence of high-spin Fe (III) and are less asymmetric as a result of the occurrence of fast spin–spin relaxation time, and their surface composition is complex, showing the presence of a multiplicity of iron oxidation and spin states (although with a majority of high-spin Fe^3+^ ions). In comparison, the β-hematin crystals prepared in aqueous-oily medium have significantly smaller lengths (ca. 560 nm) and slightly larger unit cell volume in comparison to the previous sample. The magnetic measurements show that they are affected by superparamagnetism and paramagnetism at 298 K and the coexistence of weakly ferromagnetic or possibly ferrimagnetic and paramagnetic phases at 80 K. Their Mössbauer spectra at 298 K, 77 K, and 10 K, also consistent with the presence of high-spin Fe (III), show longer spin–spin relaxation times, and their surface composition is also complex containing less surface OH^–^ groups and higher amounts of Fe (II) ions in low- and high-spin states. The observed differences are discussed in relation to the specific formation conditions present in the synthesis medium. The results reported here are of outmost importance for understanding how the physicochemical properties of β-hematins depend on the synthesis conditions.

## Introduction

The malaria disease has been recognized in human history since ancient times and it is still a major global health problem today.^1^ According to the world malaria report 2023,^2^ globally in 2022, there were an estimated 249 million malaria cases in endemic areas, an increase of 5 million cases compared with 2021. There is a permanent worldwide challenge to find proper treatments to control and eliminate this disease.

Malaria is caused when the Plasmodium parasite is transmitted to humans by the bite of a mosquito. During the life cycle of the parasite, there is a phase in which Plasmodium degrades hemoglobin, ultimately forming hemozoin crystals, also known as malaria pigment. It is now widely accepted that the hemozoin crystal is a key target to better understand the malaria disease.^3,4^ While most studies support lipid-mediated nucleation at the inner membrane surface of the parasite’s digestive vacuole, hemozoin formation can also occur without lipid catalysts, in compartments outside the digestive vacuole.^5^ Therefore, it is essential to study their formation mechanisms and physical properties in different environments. The synthetic version of the natural hemozoin is called β-hematin, and the studies on both natural and synthetic hemozoin are complementary and valuable. Despite the great efforts to understand these subjects, we notice several unsolved issues and/or controversies in the reported literature. For example, we have also observed that numerous equations, at least 11, describing the kinetics of β-hematin formation from hemin have been reported in the literature.^6^ One of the latest equations was proposed by Herrera et al.,^6^ who suggested that the biphasic nature of the kinetic curves is due to both the availability of hemin dimers and the nucleation and growth of β-hematin.

The magnetic properties of β-hematin are controversial. Inyushin et al.^7^ and Khmelinskii and Makarov^8^ reported that commercial hemozoin from InvivoGen behaves as a superparamagnet at +20 °C and −20 °C. This behavior implies that there must be ferromagnetic interactions between Fe ions within the superparamagnetic particle or region. In opposition, Giacometti et al.^9^ reported that the same commercial hemozoin sample exhibited paramagnetism, implying that no magnetic interactions exist between iron ions at these temperatures. Roch et al.^10^ reported that the paramagnetic properties of synthetic and natural hemozoin crystals yield clues to evaluate a low-cost instrument as a malaria diagnosis system. On the other hand, Fescenko et al.^11^ reported that both behaviors are possible. The authors used diamond magnetic microscopy on the same sample and found that 1 out of 41 nanoparticles were superparamagnetic and the rest, i.e., 40, paramagnetic. By employing state-of-the art electronic structure calculations, Ali and Oppeneer^12^ predicted a very weak antiferromagnetic exchange interaction between each of the iron heme centers at very low temperatures. At high temperatures paramagnetic behavior is expected.

Another intriguing topic is the potential presence of two phases in β-hematin. Straasø et al.^13,14^ and Marom et al.^15^ proposed the existence of a major phase, as published by Pagola et al.,^16^ and a minor phase observed in a few studies.^14^ The authors attribute this to the formation of four stereoisomeric heme dimers of Fe^3+^-PPIX: two centrosymmetric (cd1_1_ and cd1_2_) and two enantiomeric (cd2(+) and cd2(−)). The main difference between the major and minor phases of β-hematin lies in the composition of the hematin anhydride dimers present.^14^ The major phase is mainly composed of the centrosymmetric dimer cd1_1_, with the possible inclusion of the enantiomeric dimers cd2(+) and cd2(−). On the other hand, the minor phase is mainly composed of the centrosymmetric dimer cd1_2_, and although the enantiomeric dimers cd2(+) and cd2(−) could also be present in this phase, their concentration would be lower compared to the major phase. The presence of the minor phase of β-hematin, i.e., its formation, appears to be highly dependent on the synthesis procedure, which may explain its limited reporting.^14^

The room temperature Mössbauer spectrum of β-hematin has been reported as a highly asymmetric doublet and this asymmetry decreases with decreasing temperature until it becomes a symmetric doublet at liquid helium temperatures.^17,18^ But the question about if different spectral characteristics can be observed in samples prepared under different chemical conditions remains.

It is also important to investigate whether the oxidation and spin states of iron in the protoporphyrin-IX within β-hematin are homogeneous or heterogeneous between the interior and the surface. This is because the number and type of ligands on the surface may differ from those in the interior. In fact, theoretical^12,15,19−23^ and experimental studies^24−27^ have demonstrated that the axial ligand strongly determines the electronic structure and function of metalloporphyrin complexes. The most common oxidation states of iron in Fe-porphyrins (FeP) and Fe-protoporphyrin (FePP) complexes are Fe (II) and Fe (III).^12,19−27^ Iron in Fe (II) porphyrins and protoporphyrins can be stabilized in high (S = 2, four unpaired electrons), intermediate (S = 1, two unpaired electrons), and low (S = 0, no unpaired electrons) spin states. On the other hand, iron in Fe (III) porphyrins and protoporphyrins can be stabilized in high (S = 5/2, five unpaired electrons), intermediate (S = 3/2, three unpaired electrons), and low (S = 1/2, one unpaired electrons) spin states. The experimentally observed ground state spin for iron in some complexes are S = 1 for tetra-coordinated FeP, S = 5/2 for penta-coordinated FePCl (FeP with a chloride ligand) and FePOH (FeP with a hydroxyl ligand), S = 2 for penta-coordinated FePIm (FeP with an imidazole ring ligand), and S = 0 for hexa-coordinated FePImO_2_ (FeP ligated with both an imidazole ring and an O_2_ group). Recently, Sahoo et al.^24^ reported the stabilization of S = 5/2 for Fe (III) in a five-coordinate complex Fe III (TPPBr8) (OCHMe2), while Fe (III) in other six-coordinate complexes stabilized in admixed-high, admixed-intermediate and low-spin states. Most of these experimental observations have been proven by first principle theoretical calculations.

It is well documented in the scientific literature that a given compound, with the same chemical composition (*i.e*. the ratio of the number of atoms of an analyte element to the total number of atoms of all elements in a compound), can exhibit different morphologies, crystal sizes, and overall physicochemical properties. This variety of properties in a given compound are a consequence of the differing methods of preparation. In the case of β-hematin, it has been shown that it can be synthesized under widely different conditions. See for example, the introduction section of the paper by Herrera et al.^6^ One of the earliest reactive mediums used to synthesize β-hematin was acidic acetate solutions, but other reactive environments including the incorporation of long chain alcohols, and other media have been employed, etc. In spite of this knowledge, there are few reports in which a comparison of the different physicochemical properties of β-hematin prepared in differing media is discussed, and this is one of the purposes of the present work. We study the crystallographic, morphological, surface, electronic, magnetic, vibrational and Mössbauer spectral characteristics of β-hematin crystals, when these are prepared from hemin in aqueous-acetate and in aqueous-oily media. Our results point out that the physical characteristics depend on the synthesis environment. To the best of our knowledge, the following results are new and these are perhaps the main novelties of our work: (i) we supported that both paramagnetic and superparamagnetic behaviors in β-hematin are possible depending upon the environment of formation, (ii) we found weak ferromagnetism (or possibly ferrimagnetism) in combination with paramagnetism at 80 K in some samples, (iii) the Mössbauer spectral shape were different for samples originating in different environments, (iv) the electronic structures of the iron ions located at the surface of the β-hematin particles were found to be heterogeneous and not homogeneous and (v) we have provided a complete overview of some physicochemical properties originating from the use of different characterization techniques.

## Methodology

### Synthesis in Aqueous-Acetate Medium

The method of synthesis, with some modifications, is based on the one reported by Egan et al.^28^ More details are also given in Herrera et al.^6^ Initially, solutions of 0.02 M glacial acetic acid were prepared. In each synthesis, 80.0 mg of bovine hemin chloride was used. Hemin was dissolved in 40.0 mL of 0.4 M NaOH solution. The mixture was stirred at 100–130 rpm for 30 min. Subsequently, the temperature was raised to 60 °C, and 10.0 mL of the previously prepared solution of acetic acid were added. The mixing of acetic acid solution and basic hemin results in the formation of an acetate buffer at pH 4.75. The crystals were filtered, and 3 washes were performed with 10.0 mL of distilled water. The sample was subjected to a drying process for 48 h at 37 °C.

### Synthesis in Aqueous-Oily Medium

The synthesis was carried out with modifications to the procedures reported by Pasternack et al.^29^ and Herrera et al.^6^ In a first step, 80.0 mg of hemin were dissolved in a 0.4 M NaOH solution, to which 290 μL of dimethyl sulfoxide (DMSO) were added. The mixture was heated to 40 °C and stirred at 100 rpm for 30 min. Subsequently, the temperature was increased to 60 °C, and 10.0 mL of a 0.02 M acetic acid solution was added, along with 1.0 mL of octanol. The resulting solution reached a final pH of 4.75. Then, the solution was stirred at intervals of 25 min, alternating between 100 and 150 rpm, for a total of 3 h. After this time, the heating was stopped, and it was left to stand for an hour. The crystals were filtered, and 3 washes were performed with 10 mL of distilled water. The sample was subjected to a drying process for 48 h at 37 °C.

### Characterization Techniques

FTIR spectra were collected at room temperature using an IRTracer-100 infrared spectrometer (Shimadzu, Japan). The measurements employed 64 scans at a resolution of 4.0 cm^–1^, covering a wavenumber range from 4000 to 400 cm^–1^. The positions and intensities of the IR bands in each FTIR spectrum were determined through Voigt peak fitting,^30^ as implemented in the RECOIL software.^31^

XRD diffraction patterns were collected using a Panalytical X’Pert PRO MPD diffractometer with a Cu Kα X-ray source. A zero-background sample holder made of monocrystalline silicon with a cavity of 7 mm diameter and 1 mm depth was used. Measurements were taken over a 2θ range of 5° to 40°, with steps of 0.04° and a time per step of 23 s. Due to the presence of a small diffuse Bragg peak around 23.91° for the sample prepared in the aqueous-oily medium, new XRD measurements for this sample were taken over a 2θ range of 5° to 40°, with steps of 0.02° and a time per step of 46 s. Quantitative analysis of the XRD patterns for all samples was performed using the MAUD program, which combines the Rietveld method with Fourier transform analysis.^32^ According to Lutterotti,^32^ the Fourier analysis is used to convolute the crystallite size distribution functions and microstrains with instrumental broadening. This process allows the profile of the Bragg peaks to be calculated. Convolution using the fast Fourier transform helps avoid assumptions about the shape of the Bragg peaks.^32^ For the analysis we used the atomic coordinates reported for β-hematin by Pagola et al.^16^ and refined only the cell parameters and average crystallite sizes. The cif file from that paper was obtained via the Cambridge Structure Database. The average crystallite size was assumed to be isotropic, and the texture was considered arbitrary. Instrumental factors included in the refinement were incident intensity, a second-order polynomial background, and the three full widths at half-maximum (FWHM) of the line profile. We would like to note that the arbitrary texture option was applied in this analysis due to the varied particle size distribution, as it will be shown below, and random orientations of the particles within the sample holders. This randomness in orientation and particle size distribution supports the assumption of an isotropic average crystallite size, yielding reasonable fit results.

The SEM micrographs were obtained using a JEOL JSM-6490LV scanning electron microscope with magnifications of 200× , 5000×, 20000× , 30000× , and 40000×. The accelerating voltage was 20 kV.

^57^Fe Mössbauer spectra were recorded in the transmission mode at 298 K, 77 K, and 10 K using a constant acceleration spectrometer and a closed-cycle helium cryorefrigerator (Janis). The velocity scale was calibrated using a 6 μm thick α-iron foil and the isomer shifts referred to the centroid of the room temperature spectrum of α iron. The spectra showed clear relaxation line shapes and were fitted using the Blume–Tjon model^33^ as implemented in the RECOIL program.^31^ Blume–Tjon model^33^ assumes that the magnetic hyperfine field (H) fluctuates stochastically between +H and −H along the electric field gradient (EFG) *z* axis with asymmetry parameter η = 0 (i.e., an axially symmetric EFG). During the fitting we used the following constraints: (i) the hyperfine field was fixed to 55 T, because it was assumed that each ferric ion in the protoporphyrin IX has 5 unpaired electrons and that for each unpaired electron, the contact field is 11 T;^34^ (ii) the ratios of the subspectral areas of peak 1 to peak 3 (A_1_/A_3_) and peak one to peak two (A_1_/A_2_) were fixed to 3 and 2, respectively, which are the values for randomly oriented samples; (iii) the half difference of the flip frequency were fixed to 0, implying similar average dwell times along the + and − directions. Fitting parameters were the isomer shift, quadrupole shift, intrinsic Lorentzian half-width at half-maximum, average flip frequency, and total spectral area(s) of the site(s).

The magnetic properties of the samples were investigated using a Vibrating Sample Magnetometer (VSM) motor module of the Physical Property Measurement System (PPMS) Model 6000 (Quantum Design, San Diego, CA.) equipped with a superconducting magnet. Magnetization versus field curves were obtained at temperatures of 80 and 300 K, within a field range of 0–15000 Oe. Magnetization versus temperature curves were recorded at a constant magnetic field of H = 1 kOe, spanning temperatures from 10 to 300 K. A sample mass of 5 mg was introduced in polypropylene powder holder, by ensuring sample compaction, placed in a brass half-tube sample holder of 3.5 mm inner diameter and then attached to the end of the sample rod assembly and afterward inserted into the dewar. Analysis of the data was performed using Python and some libraries for fitting.

X-ray Photoelectron Spectroscopy (XPS) data were recorded under a pressure lower than 2 × 10^–9^ mbar using a PHOIBOS-150 electron analyzer (SPECS), Al Kα radiation and a constant pass energy of 20 eV. The binding energy scale was referred to as the main C–C signal of the C 1s core level spectrum which was set at 284.6 eV. In order to avoid sample degradation of the samples under the X-ray beam this was operated at 100 W, *i.e*., a fourth of the maximum possible power. Additionally, the spectra were investigated further at given collection times to monitor possible changes in the spectra envelopes. These were found to be stable and constant along all the scans used to collect the data. Thus, we are reasonably confident that, under the experimental conditions employed, the samples have not suffered degradation under the X-ray beam. Finally, all the spectra were computer fitted with the CASA-XPS software using Pseudo-Voigt line profiles (70% Gaussian/30% Lorentzian) and a Shirley background.

## Results and Discussion

### FTIR

**Figure 1:**
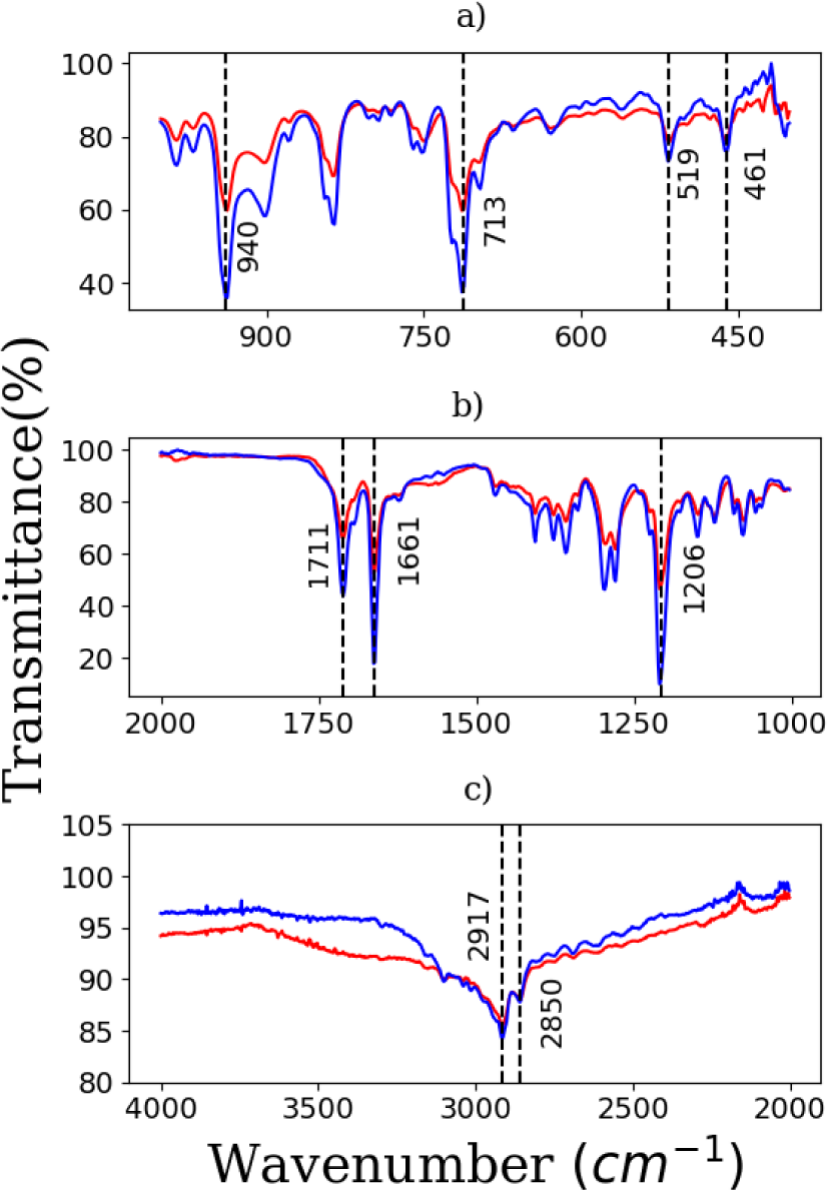
FTIR spectra recorded from samples synthesized in aqueous-acetate (blue) and aqueous-oily media (red). The spectra are divided into three spectral regions: (a) 400–1000 cm^–1^ (upper); (b) 1000–2000 cm^–1^ (middle); and (c) 2000–4000 cm^–1^ (lower).

Figure 1 shows the FTIR spectra recorded from samples synthesized in aqueous-acetate and aqueous-oily media. The spectra are divided into three spectral regions: 400–1000 cm^–1^ (Figure 1a); 1000–2000 cm^–1^ (Figure 1b); and 2000–4000 cm^–1^ (Figure 1c). To quantitatively determine the positions and intensities of the IR bands we fitted each band in the FTIR spectrum with the Voigt function using the RECOIL software.^31^ It is noted that the positions of the bands are quite similar for both samples, demonstrating that both methods of synthesis produce β-hematins crystals. In the first region (Figure 1a), the positions of some intense bands are indicated. The second region (Figure 1b) contains the principal bands which are characteristic of β-hematin formation. The band located at 1711 cm^–1^ is ascribed to the carboxylate group that is hydrogen-bonded to the second β-hematin dimer.^12^ The band located at 1661 cm^–1^ is assigned to the C=O stretch of the carboxylate group coordinated to Fe (III) center, whereas the band at 1206 cm^–1^ arises from the C–O (−Fe) single bond stretch.^12,35^ In the third region (Figure 1c), two intense bands at 2917 and 2850 cm^–1^ are observed, which are ascribed to amine groups with stretching type vibrations.^6^ Here, it is worth mentioning that several authors have employed the characteristic FTIR bands (1206, 1661, and 1711 cm^–1^) to identify β-hematin and hemozoin. For example, Chen et al.^37^ reported that *Haemoproteus* and *Schistosoma* synthesize heme polymers similar to *Plasmodium* hemozoin and β-hematin by identifying these characteristic FTIR bands. Wood et al.^36^ compared the FTIR spectra of hemozoin from mature pigmented *P. falciparum* parasites and β-hematin and identified these three bands. Other authors also used these bands to identify β-hematin.^38,39^

The FTIR analysis further confirms the absence of both B-hematin and R-hematin in the samples. As reported by Blauer and Akkawi,^40,41^ B-hematin is characterized by a strong band at 1648 cm^–1^, while β-hematin exhibits a distinct band at 1663 cm^–1^. Additionally, R-hematin is identified by prominent infrared bands near 1625 and 1224 cm^–1^.^42,43^ None of these signature bands were detected in the FTIR spectra of our samples (refer to Figure 1b and Table 1), thereby confirming that only β-hematin was synthesized.

**Table 1:** Analysis of the Complete FTIR Spectrum from 400 to 4000 cm^–1^ Becomes More Manageable when Divided into Three Distinct Regions: 400–1000 cm^–1^, 1000–2000 cm^–1^, and 2000–4000 cm^–1^a^a^

Position	Vibrational mode	Relative srea. Aqueous-acetate medium	Relative srea. Aqueous- oily medium	Discrepancies
461	Out-of-plane and metal–ligand modes	0.17	0.62	Small differences in the relative areas
519	Out-of-plane and metal–ligand modes	0.28	0.13	Small differences in the relative areas
713	Pyrrole-breathing and deformation modes	0.99	2.82	Higher relative area value for sample prepared in the aqueous-oily medium
940	-	1	1	
1206	esters/stretching/C–O (Fe)	1	1	
1661	carboxyl/stretching/link between porphyrin rings	0.36	0.50	Small differences in the relative areas
1711	carboxyl/stretching/link between dimers	0.41	0.08	Lower relative area value for sample prepared in the aqueous-oily medium
2850	amines/stretching/N–CH2	0.34	0.77	Small differences in the relative areas
2917	amines/stretching/N–CH2	1	1	

a Furthermore, this segmentation also simplifies the fitting process. Here, we present the FTIR band positions, corresponding vibrational modes, and relative areas within three spectral ranges for samples synthesized in both media. In the range from 400 to 1000 cm^–1^, the areas of bands located at 461, 517, 713, and 940 cm^–1^ are relative to the area of the band at 940 cm^–1^. In the range from 1000 to 2000 cm^–1^, the areas of bands at 1206, 1661, and 1711 cm^–1^ are with respect to the area of the band at 1206 cm^–1^. Finally, in the range from 2000 to 4000 cm^–1^, the areas of bands at 2850 and 2917 cm^–1^ are with respect to the area of the band at 2917 cm^–1^.

Now, the relative areas were determined with respect to the most intense band in each spectral region. The results are presented in Table 1. It is noticed that the relative intensities of some bands are affected by the synthesis media. The interpretation of these changes is rather difficult due the different factors that can affect the intensity such as the change of dipole moment during vibration, the concentration of the species, and the molecular environment. And additional contributions from bond strength, mass of atoms, polarizability, vibrational mode coupling, and the optical path length. In spite of this, in general, the observed discrepancies in relative areas could be ascribed to changes in particle morphology (such as the presence of nanoparticles) which may affect the relative intensity of bands, as some of them are strongly dependent on the surface-to-bulk ratio. This would support the observations found in XRD and SEM below.

### XRD

**Figure 2:**
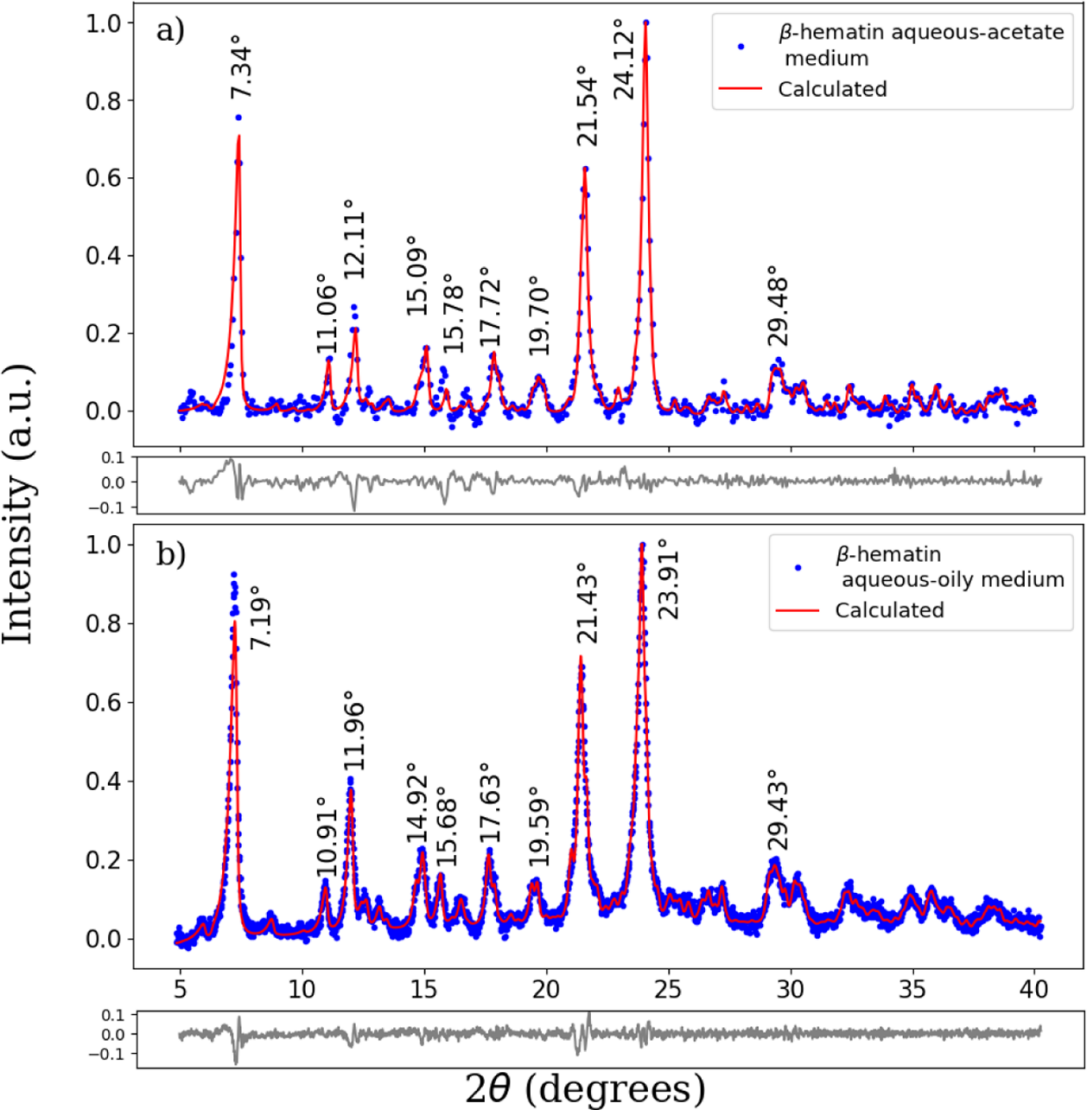
XRD patterns of β-hematins synthesized in (a) aqueous-acetate (adapted from Herrera et. al^6^) and (b) aqueous-oily media. The blue filled circles and the red lines represent the experimental XRD data and the calculated pattern using the RIETVELD method in the MAUD program, respectively. The positions of some Bragg peaks are indicated. The residuals are shown below each pattern.

**Figure 3:**
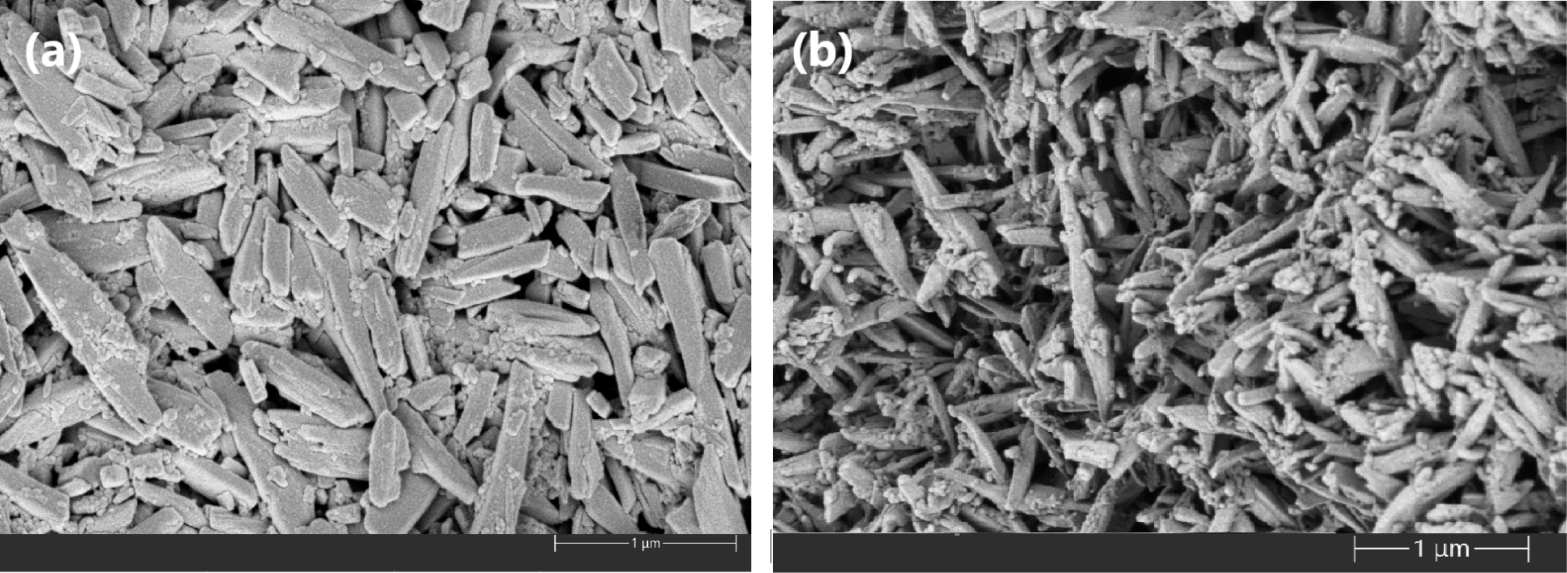
(a) SEM microphotograph of β-hematin synthesized in aqueous-oily medium. (b) SEM microphotograph of β-hematin synthesized in aqueous-acetate medium. Another microphotograph for this sample is also shown in Figure 14 of the paper by Herrera et al.^6^

Figure 2a,b shows the XRD patterns of β-hematins synthesized in aqueous-acetate and aqueous-oily media, respectively. The positions of some Bragg peaks in terms 2θ (degrees) and *Q* (1/Å) for both samples and the corresponding Miller indices are listed in Table 2. We fitted the XRD patterns using the RIETVELD method as implemented in the MAUD program.^32^ The XRD patterns calculated using β-hematin (indexed to a triclinic unit cell, P1 space group, using the atomic coordinates reported for β-hematin by Pagola et al.^16^ and refining the unit cell parameters and average crystallite sizes) closely follow the experimental data. These results are in good agreement with the FTIR findings. Now, it can be noticed that in the bottom of Figure 2b there is a small diffuse peak around the most intense peak located at 23.91°, with Miller indices of (131), perhaps indicative of the formation of an amorphous phase in the sample prepared in the aqueous-oily medium. This could indicate the possible presence of nanosized or amorphous β-hematin, that coprecipitated with the β-hematin crystals. It is probable that this idea be further supported by the SEM micrograph (Figure 3) in which it is noticed the formation of small agglomerations around the large particles with needle-like morphologies. Here it is worth mentioning that Oliveira and coworkers^44^ reported that the synchrotron radiation X-ray diffraction pattern (SR-XRD) obtained from *S. mansoni* hemozoin (Hz) displayed two contributions coming from the Bragg diffraction peaks of hemozoin crystals as well as an amorphous background signal assigned to the possible presence of either lipids or amorphous Hz pigments. Bohle and coworkers^45^ previously reported a similar diffuse scattering background effect in SR-XRD measurements of malaria pigment in red blood cells infected with *Plasmodium falciparum*. In order to check for this possibility, we have fitted again the XRD pattern of the β-hematin prepared in the aqueous-oily medium by introducing two β-hematins which differs only in their average crystallite-sizes (figure not shown): one β-hematin with an initial average crystallite size of 1000 Å was used to simulate the large brick-like particles observed in the SEM micrographs, while another β-hematin with an initial average crystallite size of 500 Å was used to represent the amorphous phase. During the fitting process, the crystallite sizes were treated as free parameters, resulting in final values of 1499 Å for the larger crystallites and 1002 Å for the smaller ones. In comparison to the first fit, which used only a single β-hematin component, the results for the total fit that uses two β-hematins of different crystal sizes showed no significant improvements either visually or in the statistical fit values. This amorphous form is probably present in such small amounts (as also suggested by SEM) and does not produce appreciable improvement in the fits. Therefore, the fitting method used, which is for a single average β-hematin component, is not in contradiction with the idea that an amorphous β-hematin is also present simultaneously with the β-hematin of large crystals.

**Table 2:** Positions of Some Bragg Peaks in Terms 2θ (Degrees) and *Q* (1/Å) for Both Samples and the Corresponding Miller Indices^a^

Sample	Aqueous-acetate medium		Aqueous-oily medium	
Miller Indices	2θ [deg]	Q [1/Å]	2θ [deg]	Q [1/Å]
100	7.34	0.52	7.19	0.51
001	11.06	0.78	10.91	0.77
020	12.11	0.86	11.96	0.85
120	15.09	1.07	14.92	1.05
111	15.78	1.12	15.68	1.11
1–21	17.72	1.25	17.63	1.25
121	19.70	1.39	19.59	1.38
031	21.54	1.52	21.43	1.51
131	24.12	1.70	23.91	1.69
212	29.48	2.07	29.43	2.07

a Here Q = (4π/λ)sin(2θ/2), where λ is the X-ray wavelength.

The unit cell parameters for both samples derived from the fits are presented in Table 3. These values are compared with the parameters reported for synthetic^16^ and natural^5^ hemozoin.

**Table 3:** Crystallographic Unit Cell Parameters Derived from the Rietveld Analysis of the XRD Patterns Using the MAUD Program^a^

Unit cell parameters	Aqueous-oily [This work]	Aqueous-acetate^6^	Pagola et al.^16^	Klonis et al.^5^
a (Å)	12.27(1)	12.23(1)	12.2	12.187(2)
b (Å)	14.81(1)	14.78(1)	14.7	14.692(2)
c (Å)	8.08(1)	8.09(9)	8.0	8.030(1)
α (deg)	90.26(9)	90.67(1)	90.2	90.94(1)
β (deg)	96.87(9)	96.56(9)	96.8	96.99(1)
γ (deg)	97.88(8)	98.23(9)	97.9	96.81(1)
V (Å^3^)	1445(2)	1438.4(4)	1416	1416.33

a Comparison with parameters reported in the literature.

Now, by carefully inspecting the XRD patterns of all samples, variations in relative intensities and peak broadening in some Bragg reflections can be observed. Solomonov et al.^46^ determined the FWHM for different Bragg reflections and, using the Scherrer formula, calculated the relative change in crystal coherence length along the *c* axis, L{001}, relative to other crystallographic directions, including {031} and {131}. To quantify these variations, we measured the areas (see Table 4) and full width at half-maximum (FWHM) (see Table 5) of the five most intense and well-resolved Bragg peaks for each XRD pattern. Regarding the intensity ratios, it is noted that *I*_001/031_, *I*_001/031_ and *I*_001/031_ are slightly larger for β-hematin crystals grown in aqueous-acetate medium, in contrast *I*_100/031_, *I*_100/031_ and *I*_100/031_ are slightly lager for β-hematin crystals grown in aqueous-oily medium. On the other hand, except for *L*_001/031_ all other relative mean coherence lengths are larger for β-hematin crystals grown in aqueous-acetate medium. These results suggest that the morphologies of the β-hematin crystals are affected by the environment of formation. As presented below, these results align well with SEM observations, which indicate that β-hematins synthesized in an aqueous-acetate medium form larger and thinner particles compared to those grown in an aqueous-oily medium.

**Table 4:** Ratios of the Intensities of (001) and (100) Bragg Peaks Relative to (020), (031) and (131) Bragg Peaks for the Different Samples^a^

Intensity ratios	*I*_001/020_	*I*_001/031_	*I*_001/131_	*I*_100/020_	*I*_100/031_	*I*_100/131_
Aqueous-acetate^6^	0.55	0.23	0.18	2.28	0.97	0.76
Aqueous-oily	0.36	0.21	0.15	2.44	1.34	0.93

a The intensity ratios *I*_(001)_/*I*_(hkl)_ and *I*_(100)_/*I*_(hkl)_ are denoted as *I*_001/hkl_ and *I*_100/hkl_ respectively. Comparison with intensity ratios reported in the literature.

**Table 5:** Ratios of the Mean Coherence Lengths *L*_*001*_ and *L*_*100*_, with Respect to the Mean Coherence Lengths *L*_*020*_, *L*_*031*_, and *L*_*131*_ for the Different Samples ()^a^

Mean length ratios	*L*_001/020_	*L*_001/031_	*L*_001/131_	*L*_100/020_	*L*_100/031_	*L*_100/131_
Aqueous-acetate^6^	0.83	0.83	0.67	1.46	1.45	1.16
Aqueous-oily	0.79	1.08	0.50	0.71	1.28	0.36

a The mean coherence length ratios *L*_(001)_/*L*_(hkl)_ and *L*_(100)_/*L*_(hkl)_ are denoted as *L*_001/hkl_ and *L*_100/hkl_ respectively.

Finally, we investigated the presence of two phases in β-hematin due the formation of four stereoisomeric heme dimers of Fe (III)-PPIX: two centrosymmetric, cd1̅_1_ and cd1̅_2_ and two enantiomeric, cd2(+) and cd2(−),^13−15^ According to the literature,^13,14^ the presence of five characteristic peaks: (i) one on the left side, like a shoulder of the (100) peak (located at about 2θ ∼ 6.6° or *Q* ∼ 0.469 Å^–1^), (ii) a second between the (1̅10) and (110) peaks (2θ ∼ 9.5° or *Q* ∼ 0.675 Å^–1^), (iii) a third between the (001) and (020) peaks (2θ ∼ 11.7° or *Q* ∼ 0.831 Å^–1^), (iv) a fourth between the (031) and (131) peaks (2θ ∼ 22.8° or *Q* ∼ 1.612 Å^–1^), and (v) a fifth on the right side of the (131) peak (2θ ∼ 25.4° or *Q* ∼ 1.793 Å^–1^), are indicative of the presence of the minor secondary phase. The main phase is that reported by Pagola et al.^16^ However, by simple visual inspection of the XRD patterns of the present samples we could not identify the presence of these five peaks assigned to the minor phase. Our results suggest that our β-hematin crystals are mainly composed of a mixture of centrosymmetric cd1̅_1_ and enantiomeric cd2(±) stereoisomers.

### SEM

Figure 3a,b displays the microphotographs taken from β-hematin samples synthesized in aqueous-oily and aqueous-acetate media, respectively. These exhibit a homogeneous distribution of particles with similar morphologies, characterized by a needle-like shape and parallelepipeds with sizes of 767 ± 64 nm for the aqueous-acetate medium and 562 ± 34 nm for the aqueous-oily medium. Both morphologies resemble those found in hemozoin, as reported by other authors,^47^ who describe the particles as flat needles. However, the change in the synthesis medium clearly results in different particle sizes, with smaller particles corresponding to the samples produced in the aqueous-oily medium. By using media that mimic the digestive vacuole, it is expected that the particles will exhibit properties similar to those of natural hemozoin,^48^ as these biomimetic conditions favor such crystallization. The particle sizes of β-hematin obtained in the aqueous-oily medium are like those of natural hemozoin.

#### Mössbauer Spectroscopy

**Figure 4:**
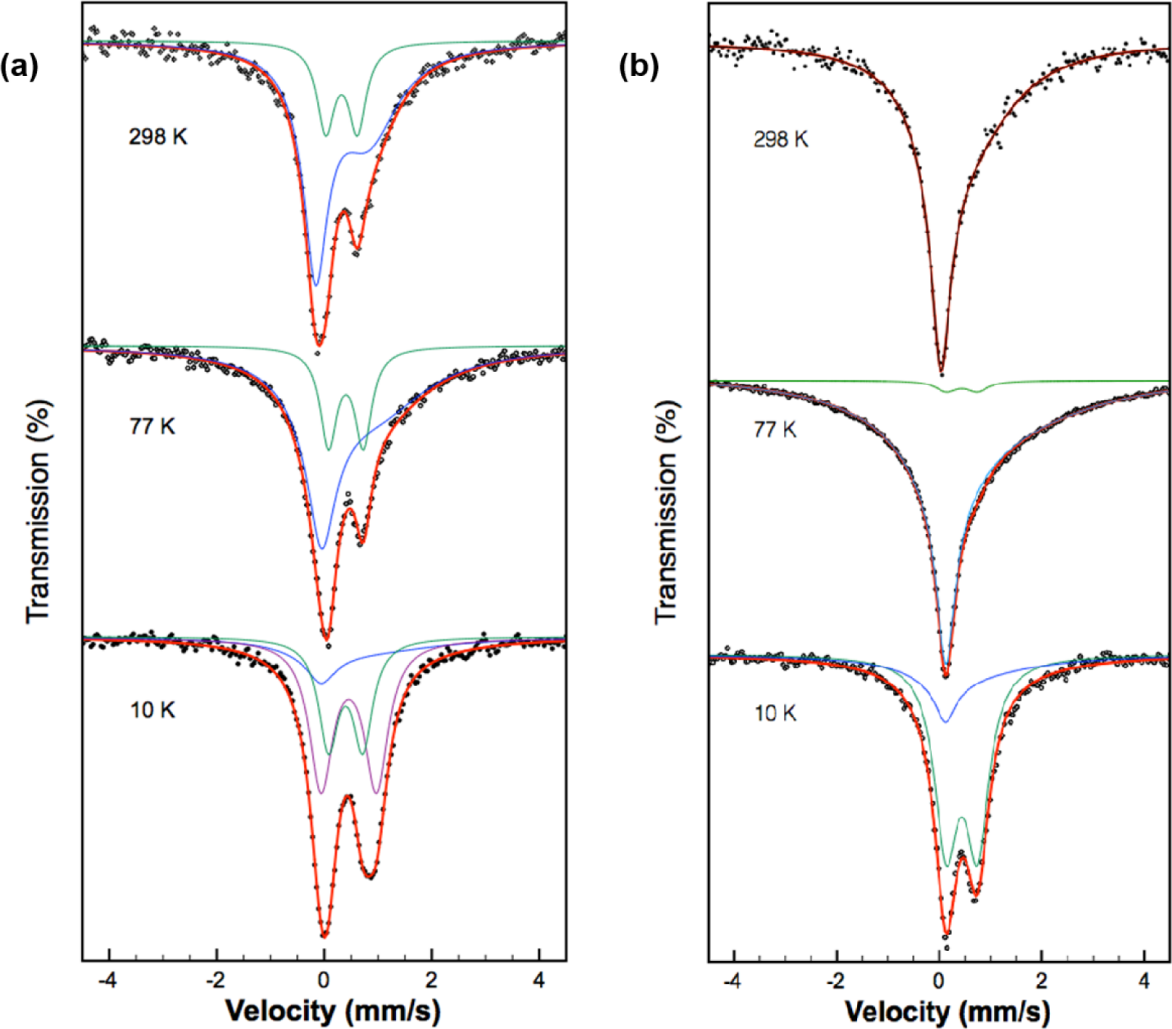
298 K (upper), 77 K (middle) and 10 K (lower) Mössbauer spectra of β-hematin synthesized in (a) aqueous-acetate medium and (b) aqueous-oily medium. The black points represent the experimental data, the red lines are the total calculated spectra, and the blue, green and magenta lines represent the subspectral components used to fit the experimental spectra.

Figure 4a,b shows the 298 K, 77 K, and 10 K Mössbauer spectra recorded from β-hematin synthesized in aqueous-acetate and aqueous-oily media, respectively. In general, they are composed of a doublet and a very asymmetric component.

Prior to embarking on a quantitative analysis of the Mössbauer spectra, it is advisible to develop a broad qualitative understanding of their principal characteristics. For that purpose, we have taken into consideration the works by Bearden et al.^49^ and by Adams et al.^17^ Bearden et al.^49^ who investigated the Mössbauer spectra of hemin and reported that the relative areas of the two lines of the doublet were equal at all temperatures, but the difference in the widths of the two lines increased with increasing temperature. Adams et al.^17^ proposed four types of spectra for ferriprotoporphyrin IX complexes: type (1): asymmetric spectra with a high-velocity line almost disappeared; type (2): asymmetric spectra with a broad and well-defined high-velocity line; type (3): symmetric spectra, for which the two lines of the doublet broadens similarly; and type (4): the high-velocity line shows a gradation intermediate between those of type (1) and type (2) spectra.

Based upon this criterion, the 298 and 77 K Mössbauer spectra of β-hematin prepared in the aqueous-oily medium can be classified as type (1). In contrast, the 298 and 77 K Mössbauer spectra of β-hematin prepared in the aqueous-acetate medium can be classified as type (4). Therefore, the qualitative spectral characteristics of β-hematin at 298 and 77 K, are very dependent on the synthetic medium.

The Mössbauer spectra at 10 K for both samples are classified as type (2). No spectra could be classified as type (3), *i.e*., symmetric spectra. Therefore, for a given β-hematin, the spectrum collected at 10 K is more symmetric than the spectra collected at 298 and 77 K.

The previous analysis is based upon simple and qualitative classification of the spectral shapes as proposed by Adams et al.^17^ However, a more quantitative analysis requires the use of proper fitting models. Stanek and Dziedzic-Kocurek^18^ highlighted that characterizing the Mossbauer spectra of diluted paramagnetic Fe^3+^ ions in organic materials, as it is for β-hematin, is among the most challenging aspects of Mossbauer spectroscopy. Blume^34^ attributed the asymmetry in the Mössbauer spectra of hemin (FeIII-protoporphyrin) to temperature dependent electronic spin–spin relaxation. The author suggested that the relaxation process decelerates as temperatures rise, influenced by changes in the population of the ±5/2, ± 3/2, and ±1/2 electronic energy levels. Therefore, the Mössbauer spectrum is a superposition of relaxation spectra coming from these three electronic levels. However, very similar spectral shapes can be obtained by using a simplified relaxation model, which takes only into account the relaxation in the ±5/2 energy levels. This simplified relaxation behavior, which was proposed by Blume and Tjon,^33^ assumes that the magnetic hyperfine field (H) fluctuates stochastically between +H and −H along the electric field gradient (EFG) Z axis with asymmetry parameter equal to cero. This so-called Blume-Tjon model was used in the analysis of the Mössbauer spectra for all samples as it is implemented in the RECOIL program.^31^ The spectra were fitted with one, two or up to three components. The 298 K spectrum for β-hematin synthesized in aqueous-acetate medium requires two spectral components (asymmetric and symmetric doublets) for a proper fitting, whereas the 298 K spectrum for β-hematin synthesized in aqueous-oily medium required only one component, an asymmetric component, which is not strictly a doublet but a sextet affected by spin–spin relaxation. Since the line appearing at most positive velocities in the 10 K spectrum of the former clearly broadens it was necessary to include a third component in the fit, so the spectrum was fitted using an asymmetric component and two symmetric doublets. For the latter, however, only two components (one symmetric and one asymmetric) were needed. As the temperature decreases, the spectral area of the asymmetric doublet diminishes, while the area of the symmetric doublet increases. This behavior can be explained by using Blume’s model.^34^ At low temperatures, only the ±1/2 levels, which have high relaxation rates, are occupied and therefore symmetric spectra are expected. As the temperature increases, the excited levels of the Fe^3+^ ion become populated. These excited levels have slower relaxation rates. ^57^Fe nuclei whose ions are in these states produce asymmetric spectra. As the temperature continues to increase, the ±1/2, ± 3/2 and ±5/2 levels are equally occupied.

The hyperfine parameters derived from the fits of the 298 K, 77 K, and 10 K Mössbauer spectra of β-hematin synthesized in aqueous-acetate medium are presented in Table 6, while, the hyperfine parameters derived from the fittings of the 298 K, 77 K, and 10 K Mössbauer spectra of β-hematin synthesized in aqueous-oily medium are presented in Table 7.

**Table 6:** Hyperfine Parameters Derived from the Fit, Using the Blume–Tjon Relaxation Model, of the 298 K, 77 K, and 10 K Mössbauer Spectra of β-Hematin Synthesized in Aqueous-Acetate Medium^a^

Temperature (K)	δ (mm·s ^–1^)	Δ (mm·s^–1^)	τ (ns)	Area (%)
298	0.33	0.96	1.20	79
	0.32	0.60	-	21
77	0.47	1.02	11.0	69
	0.40	0.64	-	31
10	0.47	1.06	8.04	22
	0.40	0.64	-	29
	0.46	1.02	-	49

a The isomer shift δ is specified relative to metallic iron at ambient temperature and was not corrected for the second-order Doppler shift. Δ is the quadrupole splitting, τ is the relaxation time of the local magnetic hyperfine field (Bhf) within the framework of the Blume–Tjon relaxation model, and A is the spectral area of the component. Due to overlapping of the spectral components, estimated errors are of about ±0.02 mm/s for δ, ± 0.02 mm/s for Δ, ± 0.02 ns for τ, and ±5% for areas.

**Table 7:** Hyperfine Parameters Derived from the Fit, Using the Blume–Tjon Relaxation Model, of the 298 K, 77 K, and 10 K Mössbauer Spectra of β-Hematin Synthesized in Aqueous-Oily Medium^a^

Temperature (K)	δ (mm·s^–1^)	Δ (mm·s^–1^)	τ (ns)	Area (%)
298	0.33	0.60	1.98	100
77	0.47	0.69	4.80	98
	0.44	0.62	-	2
10	0.42	0.60	6.32	30
	0.44	0.62	-	70

a The isomer shift δ is specified relative to metallic iron at ambient temperature and was not corrected for the second-order Doppler shift. (Δ) is the quadrupole splitting, τ is the relaxation time of the local magnetic hyperfine field (Bhf) within the framework of the Blume–Tjon relaxation model, and A is the spectral area of the component. Due to overlapping of the spectral components, estimated errors are of about ±0.02 mm/s for δ, ± 0.02 mm/s for Δ, ± 0.02 ns for τ, and ±5% for areas.

We have found that the Mössbauer spectral shape changes in a complex manner with temperature and synthetic medium. Moreover, it is noted that two (or three) highly overlapping spectral components were required to fit the Mössbauer spectra at low temperatures, even for those samples that at 298 K were fitted with a single component. The origin of these two spectral components is difficult to interpret. Two spectral components, symmetric and asymmetric, were reported in previous works by Bauminger et al.,^43^ in a sample designated R (regular)-hematin, and by Stanek and Dziedzic-Kocurek,^18^ in a sample containing a mixture of porphyrin monomers (commercial ferriprotoporphyrin IX chloride) and μ-oxo dimers. In both works, the symmetric component is assigned to dimeric units of ferriprotoporphyrin IX antiferromagnetically coupled, whereas the asymmetric component is due to slow spin–spin relaxation. In the case of our samples, the variation in the iron–iron distances are partly responsible for the diverse shapes of the spectra. The more symmetric-like spectral component can be assigned to closer Fe^3+^-Fe^3+^ distances whereas the more asymmetric-like component can be related to larger Fe^3+^-Fe^3+^ distances. This interpretation is supported by previous works by Lang et al.^50^ and Adams et al.^17^ Lang et al.^50^ studied the Mössbauer spectra of Fe^3+^-PPIX (chloride) at 4.2 K, both in the absence and presence of an applied external magnetic field, and demonstrated that the relaxation rates decrease when the Fe–Fe distances increase, such as when Fe^3+^-PPIX is diluted in tetrahydrofuran. Adams et al.^17^ investigated the electronic environment of iron in heme and various Fe^3+^-PPIX-antimalarial complexes using 78 K Mössbauer spectroscopy, and identified four distinct types of spectra, from highly asymmetric to fully symmetric. The more symmetrical Mössbauer spectra was associated with faster relaxation rates and closer iron–iron distances.^17^

^57^Fe Mössbauer spectroscopy directly detects the nuclear transitions of the ^57^Fe, whose energy levels are sensitive to the electronic environment surrounding the iron ions. We explicitly assume that the iron ions neither change their oxidation states of 3+ nor their high spin states of 5/2. We note that the hyperfine field was fixed to 55 T, because it was assumed that each ferric ion in the protoporphyrin IX has 5 unpaired electrons and that for each unpaired electron, the contact field is 11 T. The ratios of the subspectral areas of peak 1 to peak 3 (A_1_/A_3_) and of peak one to peak two (A_1_/A_2_) were fixed to 3 and to 2, respectively, which are the values for randomly oriented samples.

At any of the given temperatures, i.e., 10 K, 77 K, or 298 K, the isomer shift, δ, values did not change with the variation of synthetic medium. The most important piece of information that can be retrieved from the isomer shifts is that the values (δ values in the range of ∼0.1 to ∼0.6 mm/s) are consistent with trivalent iron ions in high spin states. The isomer shift increases with decreasing temperature. This behavior can be explained by the second order Doppler shift, δ_SOD_. To understand this, we can use the Debye temperatures, θ_D_, reported by Dziedzic-Kocurek et al.^51^ for dimers and monomers. For the dimers with θ_D_ = 166 K,^51^ we have at 298 K that δ_SOD_ ∼ −0.22 mm/s, whereas at 10 K, δ_SOD_ ∼ −0.05 mm/s. On the other hand, for the monomers with θ_D_ = 150 K,^51^ we have at 298 K, that δ_SOD_ ∼ −0.22 mm/s; at 10 K δ_SOD_ ∼ −0.04 mm/s.

The values of the quadrupolar splittings, (Δ), can be explained using the paper by Rafiee and Hadipour.^52^ The quadrupolar interaction is sensitive both to the charge density and to the symmetry of the electric field gradient (EFG) around the ^57^Fe nucleus. Rafiee and Hadipour^52^ reported that in β-hematin, the charge distribution density plays a major role in comparison to the symmetry of EFG, and that therefore the component with lower Δ*v*alue can be assigned to those iron ions located where lower charge density could be expected.

As noted, Mössbauer spectroscopy has provided valuable information about the interactions of the Fe ions located in the FeIII-PPIX with their surrounding electronic and atomic environment. Now, we will focus on the magnetic interactions between the magnetic entities, most likely the Fe^3+^ ions, present in the samples.

#### Magnetization Measurements

**Figure 5:**
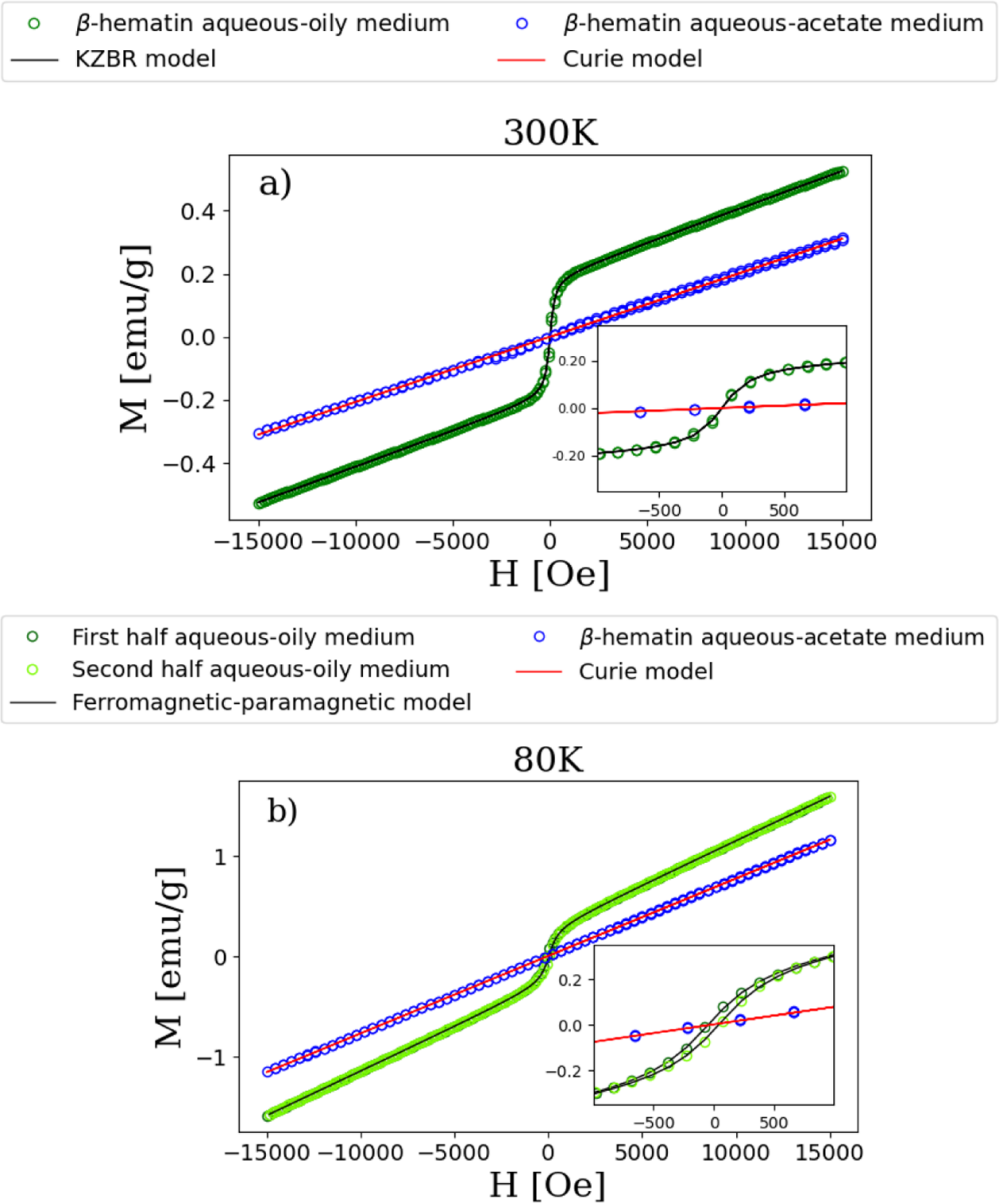
(a) Magnetization as a function of the magnetic field at 300 K for β-hematins synthesized in an aqueous-oily medium (green open circles) and an aqueous-acetate medium (blue open circles). The black and red curves correspond to the fittings using the KZBR and Curie–Weiss equations, respectively. (b) The magnetization curve as a function of the magnetic field at 80 K is shown for β-hematins synthesized in an aqueous-oily medium (green open circles) and an aqueous-acetate medium (blue open circles). The black and red curves represent the fittings using a combination of a ferromagnetic plus paramagnetic equation and a Curie–Weiss equation, respectively. The insets provide a detailed view of the magnetization curves in the central field region.

Figure 5a shows the 300 K magnetization (M) versus magnetic applied field (H) curves for both samples. The paramagnetic nature of β-hematin prepared in the aqueous-acetate medium is evident at this temperature. Therefore, the 300 K, M vs H curve for this sample fits with the Curie–Weiss model for paramagnetism,^53,54^ which is the equation of a straight line passing through the origin. The fit gives a slope value of (2067 ± 3) × 10^–8^ emu/gOe. On the other hand, the 300 K, M vs H curve for β-hematin prepared in the aqueous-oily medium exhibits a combination of both superparamagnetic and paramagnetic behaviors. Thus, the magnetization curve for this sample fit with the equation recently proposed by Kirkpatrick et al.,^55^ which consists of two terms: one linked to the superparamagnetic curve and the other representing the linear component of the paramagnetic contribution.

Equation 1, called KZBR after the names of the authors Kirkpatrick-Zhou-Bunting-Rinehart, is given by^55^
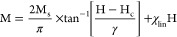
1where *M*_*s*_ denotes the saturation magnetization for the superparamagnetic component, *H*_*c*_ represents the magnetic coercivity, γ is scale parameter with magnetic field units, and χ_lin_ denotes the susceptibility of the paramagnetic component. The fit yields the following values *M*_*s*_ = (189.1 ± 0.2) × 10^–3^ emu/g; *H*_*c*_ = 0 Oe (this value was fixed); γ = 171 ± 1 Oe; and χ_lin_ = (225.6 ± 0.2) × 10^–7^ emu/gOe.

Figure 5b shows the 80 K magnetization curves for both samples. β-hematin prepared in the aqueous-acetate medium shows paramagnetic behavior at this temperature. The fitting of the 80 K, M vs H curve with the Curie–Weiss model for paramagnetism^53,54^ gives a slope value of (7726 ± 3) × 10^–8^ emu/gOe, which is higher than the value obtained at 300 K. This result is expected, as paramagnetic theory predicts that the slope of the M vs H graph is inversely proportional to temperature.^53,54^ This occurs because, as the temperature decreases, the thermal vibrational energy of the paramagnetic ions is reduced, making it easier for them to align with the applied magnetic field, thereby increasing the magnetization. Consequently, a greater slope is observed at lower temperatures. On the other hand, the 80 K, M vs H curve for β-hematin prepared in the aqueous-oily medium exhibits a combination of ferromagnetic (or ferrimagnetic) and paramagnetic behaviors. Thus, the magnetization curve for this sample was fit with eq 2:^52,56^
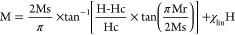
2where *M*_*r*_ denotes the remanent magnetization. The fit gives the following values: *M*_*s*_ = (273 ± 2) × 10^–3^ emu/g; *H*_*c*_ = 51 ± 2 Oe; *M*_*r*_ = (2.6 ± 1) × 10^–2^ emu/g; and χ_lin_ = (882.1 ± 0.4) × 10^–7^ emu/gOe.

In summary, the results of the magnetic measurements carried out according to Figure 5 suggest that the magnetic behavior of β-hematin depends upon the synthesis method and temperature of the measurement. At 300 K, the results clearly indicate that the sample synthesized in the aqueous-acetate medium exhibits purely paramagnetic behavior, whereas the sample prepared in the aqueous-oily medium displays a combination of paramagnetic and superparamagnetic behaviors. The last behavior suggests the presence of small regions within the crystals with internal ferromagnetic interactions. However, the weak interactions between these small regions prevent this magnetic ordering from extending throughout the material, indicating superparamagnetic behavior. At 80 K, the aqueous-acetate sample remains paramagnetic, while the aqueous-oily sample exhibits a combination of paramagnetic and ferromagnetic behaviors. The last behavior is possibly due to the interaction between these internal regions, leading to the growth of the ferromagnetic domains.

**Figure 6:**
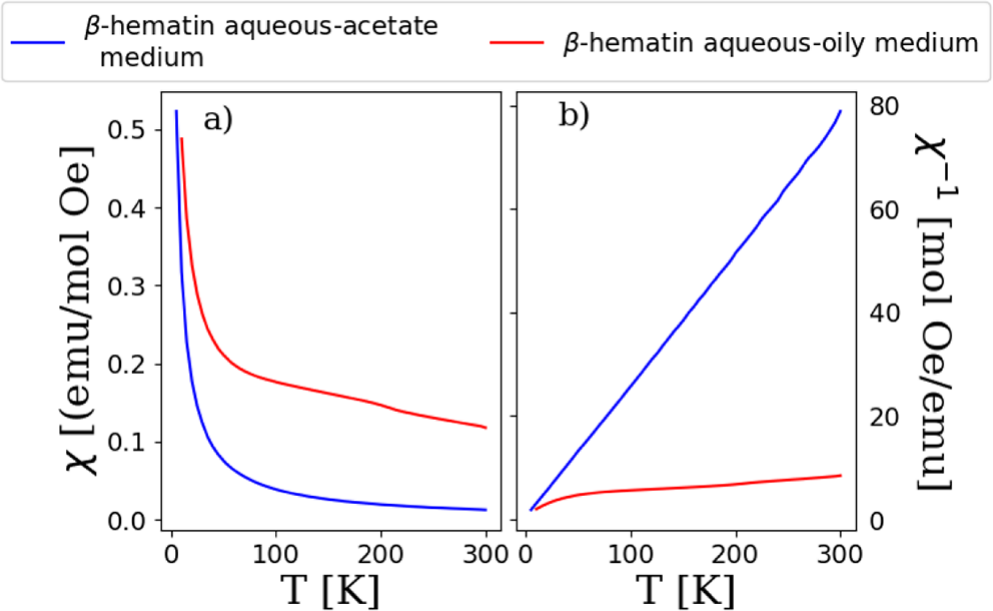
(a) Susceptibility (*χ)* versus temperature (*T*) curves for β-hematins synthesized in aqueous-oily medium (red curve) and in aqueous-acetate medium (blue curve). (b) Inverse susceptibility versus temperature curves for β-hematins synthesized in aqueous-oily medium (red curve) and in aqueous-acetate medium (blue curve).

Figure 6 shows the susceptibility (χ) and the inverse susceptibility (χ^–1^) versus temperature (T) curves for both samples. Again, the magnetic behavior is different for both samples.

The Curie–Weiss law for paramagnets is given by^53^. And therefore, the inverse of this law is given by , in which *C* is the Curie–Weiss constant and θ is a constant with temperature dimensions and indicates the strength of the interactions between magnetic moments. The value of θ allows for the calculation of the exchange integral *J*, which is a measure of the extent of electrostatic energy interaction between iron ions in the crystal. Mathematically is expressed as^54^, where *k* is the Boltzmann constant, *z* is the coordination number, and *S* is the spin angular momentum. We have considered z = 5 and S = 5/2. Table 8 lists the parameters derived from the fit using Curie–Weiss relation. The obtained *J* value is comparable with the theoretical value of *J* = 0.01 cm^–1^ that was reported by Ali and Oppeneer.^12^

**Table 8:** Parameters Derived from the Fit^a^

Parameters	Aqueous-acetate	Aqueous-oily
C (K·emu/mol·Oe)	(3891 ± 3) x 10^–3^	-
θ (K)	–0.7 ± 0.3	-
J (cm^–1^)	–0.02 ± 0.01	-

a Curie–Weiss relation.

Based on the results obtained so far, an important question arises: why does the same sample exhibit different magnetic behaviors at various temperatures? Specifically, superparamagnetic and paramagnetic behavior at 298 K; ferromagnetic (or possibly ferrimagnetic) and paramagnetic behavior at 77 K; and antiferromagnetic behavior at very low temperatures, as predicted by the Curie–Weiss law. The paramagnetic and antiferromagnetic behaviors can be explained through the advanced electronic structure calculations reported by Ali and Oppeneer.^12^ Their analysis revealed that the magnetically active orbitals undergo significant hybridization between iron 3d orbitals and σ- and π-type orbitals across the extended bridging atoms. This hybridization likely plays a key role in the temperature-dependent magnetic properties observed in the sample. The interconnected magnetically active orbitals show the presence of long-range interactions through a bond-operative exchange mechanism. The magnetically active orbitals are not solely localized on the Fe centers but result from the hybridization of d orbitals of the Fe atoms, π orbitals of O atoms, σ orbitals of bridging CH_2_, and pyrrolic π orbitals. As a combined result, a very weak antiferromagnetic coupling arises in β-hematin. In the crystalline phase, there are shorter Fe–Fe separations (7.86, 8.04, and 8.07 Å). However, these iron atoms in adjacent unit cells are not connected through exchange paths, only by weak London interactions. The superparamagnetic and ferromagnetic (or ferrimagnetic) behaviors are more difficult to explain. It is possible that this could be due to the formation of different types of dimers^5^ as well as fragmentation products^57^ that produce Fe–Fe exchange interactions in β-hematin with variable magnitude and nature. More research is required in this field.

### XPS

**Figure 7:**
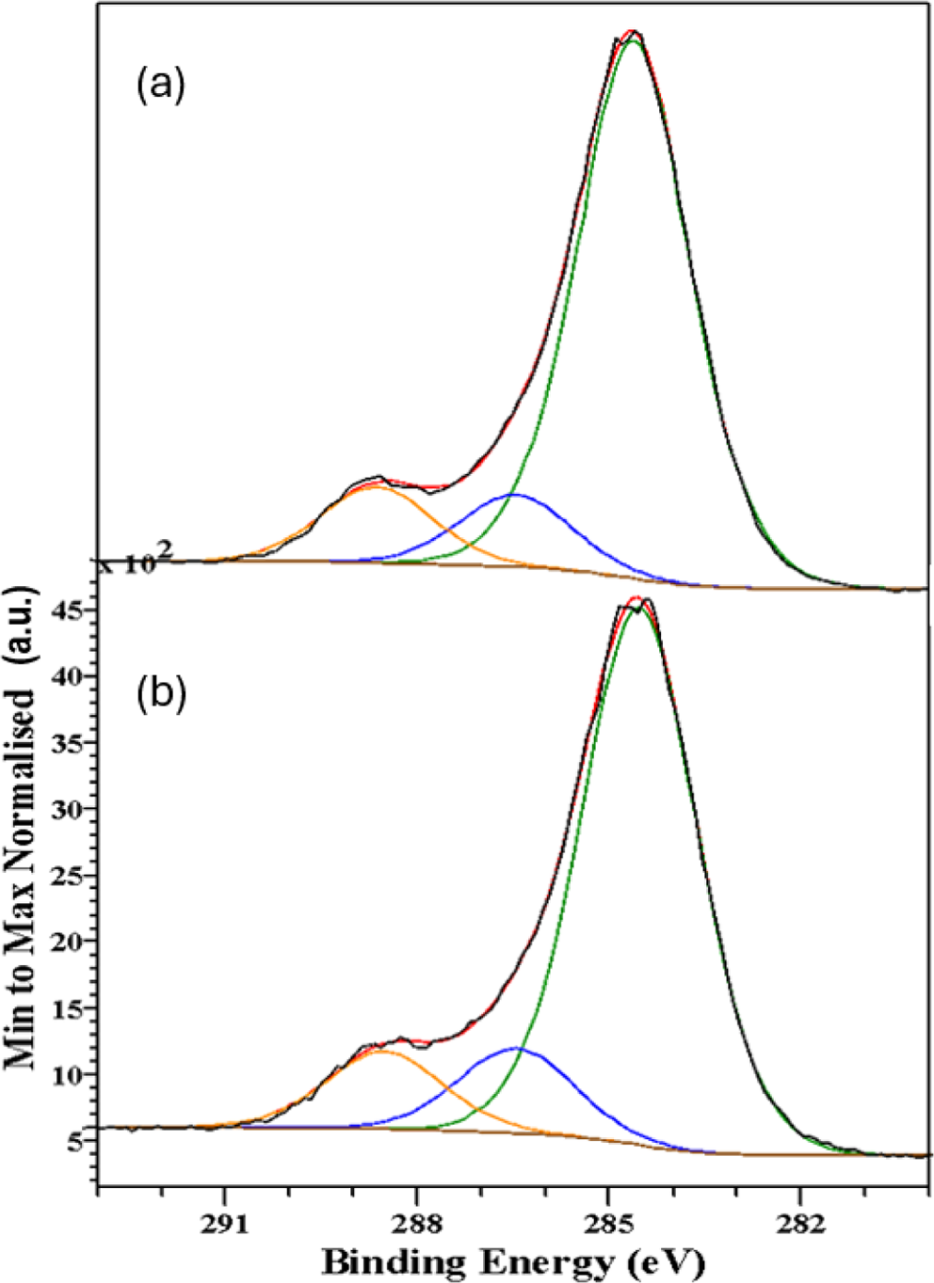
C 1s spectra recorded from the samples synthesized in (a) aqueous-oily medium, and (b) aqueous-acetate medium. The black, red, green, blue, and orange lines correspond to the experimental data, the total calculated fit, and the contributions from C–C, C–O/C–OH, and C=O/O–C=O bonds, respectively.

**Figure 8:**
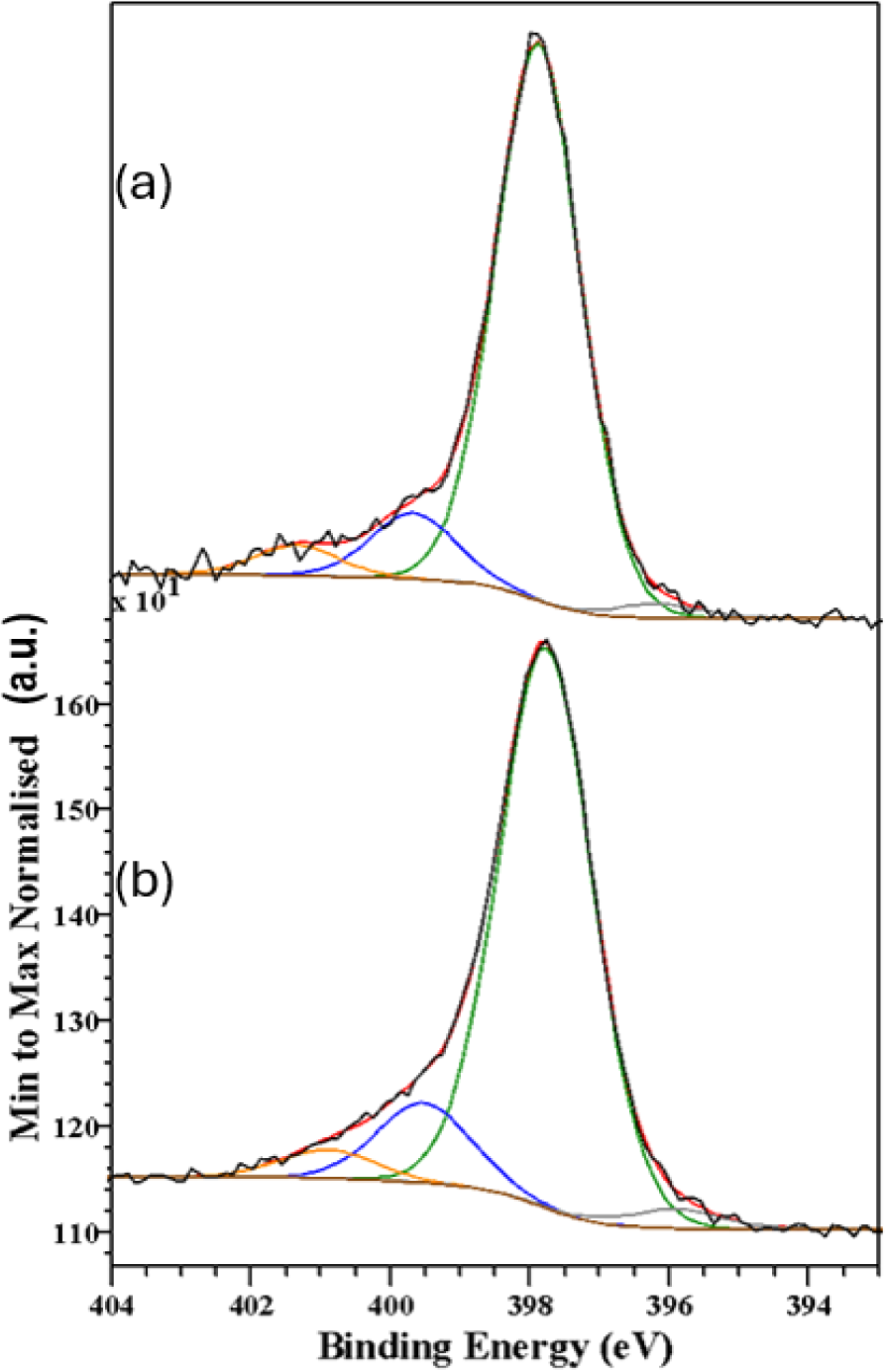
N 1s spectra recorded from the samples synthesized in (a) aqueous-oily medium and (b) aqueous-acetate medium. The black, red, purple, green, blue, and orange lines correspond to the experimental data, the total calculated fit, and the contributions from unknown, Fe–N_4_, Pyrrolic N, and π–π* assignments, respectively.

**Figure 9:**
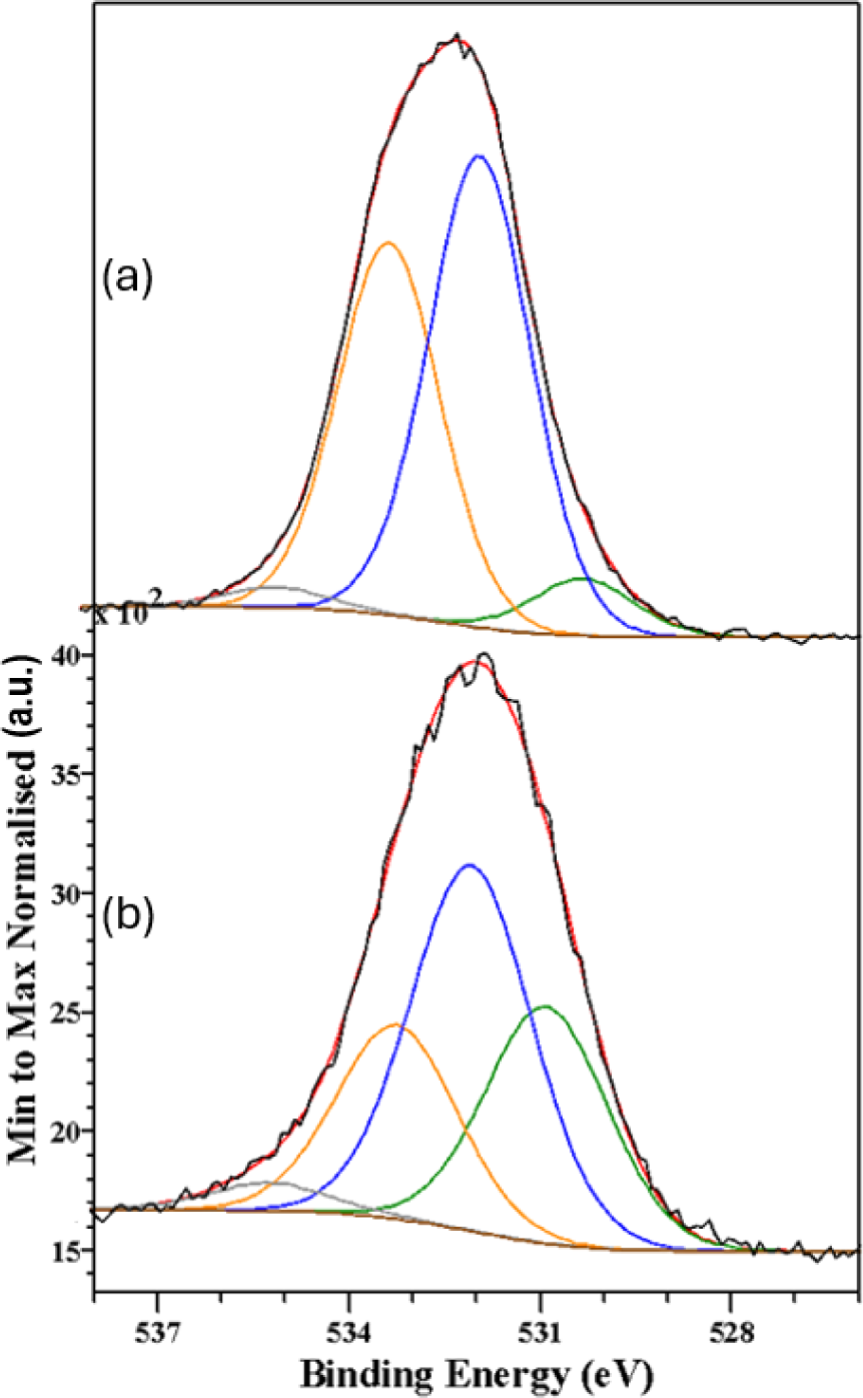
O 1s spectra recorded from the samples synthesized in (a) aqueous-oily medium and (b) aqueous-acetate medium. The black, red, green, blue, orange and purple lines correspond to the experimental data, the total calculated fit, and the contributions from OH, C=O, physisorbed water and chemisorbed water assignments, respectively.

**Figure 10:**
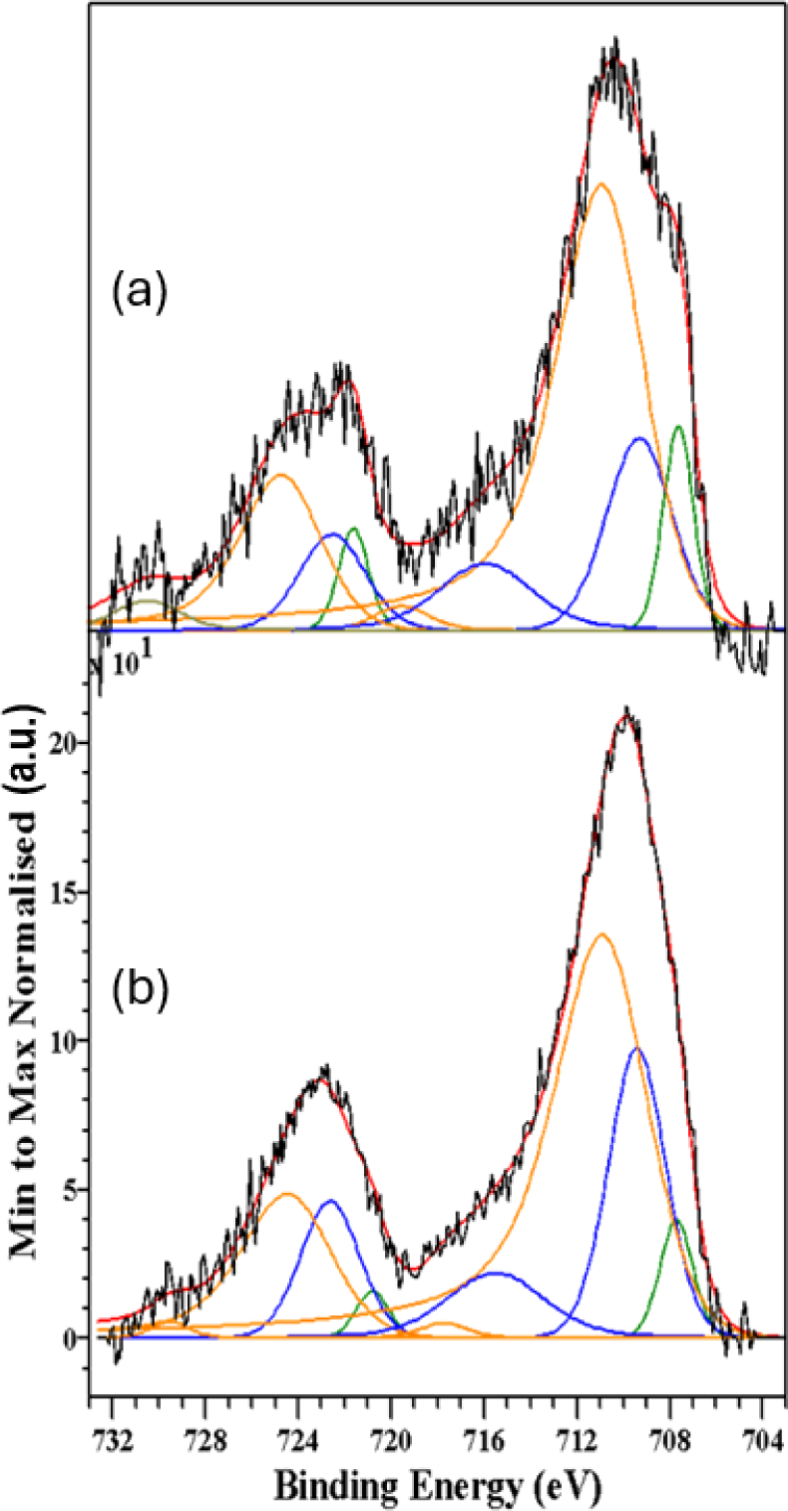
Fe 2p spectra recorded from the samples synthesized in (a) aqueous-oily medium and (b) aqueous-acetate medium. The black, red, green, blue, and orange lines correspond to the experimental data, the total calculated fit, and the contributions from low spin Fe (II), high spin Fe (II) and high spin Fe (III), respectively.

Figures 7–10 show, respectively, the C 1s, N 1s, O 1s and Fe 2p spectra recorded from the samples prepared in both media.

The C 1s spectra contain three contributions whose corresponding binding energies are collected in Table 9. The main contribution at 284.6 eV corresponds to C–C bonds, that appearing at 286.7 eV can be associated with the presence of C–OH/C–O groups and the third one located at 288.7 eV can be assigned to C=O/O–C=O bonds.^58^ The relative areas of the different contributions obtained from the fit of the spectra are also collected in Table 9. Inspection of Table 9 shows that the differences among the various samples are minimal.

**Table 9:** Binding energies, Relative Spectral Areas and Assignment of the Contributions to the C 1s Spectra

		Aqueous-oily	Aqueous-acetate
Binding energy (eV)	Assignment	Relative area (%)	Relative area (%)
284.6	C–C	78	77
286.7	C–O/C–OH	10	12
288.7	C=O/O–C=O	11	11

The N 1s spectra were fitted to four different components (Table 10). The minor one, located at 396.2 eV, has a binding energy similar to those shown by nitrogen in metal nitrides or in cyanides.^59^ The occurrence of such types of species is of difficult rationalization within the context of the present samples. This component has a very low relative area (3% in average) and it might correspond to a fitting artifact. In fact, changing the Gaussian/Lorentzian percentages of the Pseudo-Voigt profiles to 50%/50% makes unnecessary the inclusion of such contributions in the fit of some of the spectra. Its nature and occurrence are, thus, doubtful. The main contribution to the N 1s spectra appears at 398.0 eV and it accounts for 79–84% of the total spectral area. This binding energy has been associated to the metal-N_4_ species in porphyrins,^60−62^ and it would correspond to the Fe–N_4_ units present in the β-hematin molecule. The presence of the components at 399.7 and 401.3 eV, can have various assignments. That appearing at 399.7 eV has been associated with pyrrolic nitrogen,^61^ and may be arising from broken β-hematin units where iron is missing, or from the presence of sp^3^-N in nitrogen containing molecules adsorbed to the surface of the β-hematin crystals.^60^ The component at 401.3 eV has been also observed previously^60^ and has been related to the π–π* transition of the delocalized electrons, as shown for hematin anhydride. These two components together amount for approximately 15% in average of the total spectral area. Again, the N 1s spectra are very similar for all the studied samples.

**Table 10:** Binding Energies, Relative Spectral Areas and Assignment of the Contributions to the N 1s Spectra

		Aqueous-oily	Aqueous-acetate
Binding energy (eV)	Assignment	Relative area (%)	Relative area (%)
396.2	No assignment	2	3
398.0	Fe–N_4_	83	81
399.7	Pyrrolic N	10	12
401.3	π–π*	5	4

The O 1s spectra were generally broad and they were fitted to four different components assigned to OH, C=O, physisorbed water and chemisorbed water. The binding energies, relative spectral areas and assignment to different oxygen chemical species are all summarized in Table 11.^60^ Perhaps the most relevant finding is that the samples prepared in aqueous-acetate media contain a larger percentage of OH^–^ groups than their aqueous-oily counterparts.

**Table 11:** Binding Energies, Relative Spectral Areas and Assignment of the Contributions to the O 1s Spectra

		Aqueous-oily	Aqueous-acetate
Binding energy (eV)	Assignment	Relative area (%)	Relative area (%)
530.7	OH	6	29
532.0	C=O	51	44
533.5	Physi-Chemi-sorbed water	40	23
535.5		3	4

The Fe 2p spectra are surprisingly complex. According to the literature and the Mössbauer results the samples should contain exclusively high spin Fe (III) species. However, the XPS data show the presence of three different iron species (the corresponding binding energies and assignments are collected in Table 12).

**Table 12:** Binding Energies, Relative Spectral Areas and Assignment of the Contributions to the Fe 2p Spectra

			Aqueous-oily	Aqueous-acetate
Core level	Binding Energy (eV)	Assignment	Relative area (%)	Relative area (%)
Fe 2p_3/2_	710.9	High spin Fe (III)	60	59
Fe 2p_1/2_	724.6			
Satellite 1	719.5			
Satellite 2	730.7			
Fe 2p_3/2_	709.4	High spin Fe (II)	29	35
Fe 2p_1/2_	722.6			
Satellite 1	716.1			
Fe 2p_3/2_	707.7	Low spin Fe (II)	11	6
Fe 2p_1/2_	721.5			

Inspection of Table 12 indicates that the main contribution to the Fe 2p spectra arises from a spin–orbit doublet, accompanied by characteristic shake up satellites, typical of a high spin Fe (III) species^63^ as expected for β-hematin.^60^ However, the very clear presence of a satellite peak at 715–716 eV strongly suggests the concomitant presence of a high-spin Fe (II) species, hence the inclusion in the fit of the corresponding spin–orbit doublet. The binding energies for the Fe 2p_3/2_ and Fe 2p_1/2_ core level peaks of this doublet are well within the range expected for this type of chemical species.^63^ Finally, the spectra show very clearly the presence of a third narrow spin–orbit doublet whose Fe 2p_3/2_ and Fe 2p_1/2_ core level binding energies are characteristic of a low-spin Fe (II) species.^64,65^ We must recall that XPS is a surface analytical technique, that its depth probe is around 3–5 nm and that the chemical state of the surface is not necessarily representative of the chemical state of the bulk. Thus, the present results indicate that the chemistry of the surface is rather complex, and that the overall chemical situation is more intricate than that anticipated by transmission Mössbauer spectroscopy (*i.e*., a bulk analytical technique). It is generally agreed, although there are also exceptions, that the occurrence of high spin Fe (II) is related with a penta-coordinated Fe(II) center located out of the porphyrin planes, as it has been reported in Fe (II) heme derivatives.^25,26^ The occurrence of a low-spin Fe (II) species has been reported, for example, in hexa-coordinated Fe (II) centers pertaining to PPIX molecules prepared from the dissolution of β-hematin in various media and having a variety of ligands.^25,27^ It seems, then, that the surface of the present β-hematin crystals could contain a multiplicity of iron species in different spin, oxidation and coordination states resulting from either the surface degradation of β-hematin or the absorption of different ligands to the iron sites during the preparation of the samples. The results also show that the amount of extra Fe (II) species is slightly larger in the samples prepared in aqueous-oily media than in the samples prepared in aqueous-acetate media. As mentioned in the experimental section we are confident that the possible surface changes or degradation of the β-hematin crystals are not related to the irradiation of the samples under the X-ray beam. It is also important to recall that these extra Fe (II) species represent at most 40% of the total XPS spectral area; therefore, if they are located preferentially at the surface of the β-hematin crystals they represent all together only a tiny amount of the overall iron species and they could pass undetected in a transmission Mössbauer spectrum. Furthermore, the characteristic time of photoemission, which is determined by the core-hole lifetime, is around 10^–15^ s. This time is much shorter than the Mössbauer characteristic time of 10^–8^ s what implies that in XPS we see the iron species much better localized in time than in Mössbauer spectroscopy and, consequently, this small amount of Fe (II) species could be hidden behind the strong relaxation that affects the Mössbauer spectra of these systems.

Our results demonstrate that the physicochemical properties of β-hematins strongly depend on the formation environment, either aqueous-acetate or aqueous-oily. Below, we discuss how β-hematins can form from hemin in these two environments. Egan et al.^28^ reported that β-hematin formation from hematin in an acidic acetate solution follows the Avrami model, where crystallization occurs via nucleation and growth. They proposed that the acidic acetate medium acts as a phase transfer catalyst, facilitating the rapid precipitation of amorphous hematin, which then slowly transforms into crystalline β-hematin. In another study, Egan et al.^66^ found that β-hematin forms rapidly and spontaneously at long-chain alcohol/water and lipid/water interfaces under acidic physiological conditions. Similarly, Huy et al.^67^ demonstrated that alcohols promote β-hematin formation by dissociating aggregated heme into soluble monomers, reducing surface tension, and increasing supersaturation, thereby enhancing crystal nucleation and growth. The effectiveness of alcohols in this process correlates with their hydrophobicity and ability to solubilize heme. Pasternack et al.^29^ further emphasized that alcohol hydrophobicity plays a key role in β-hematin formation. More hydrophobic alcohols solubilize heme more effectively, increasing the concentration of soluble heme monomers and promoting nucleation and crystal growth. Moreover, the formation of an aqueous/non-aqueous interface, driven by hydrophobic interactions, accelerates the conversion of heme to hemozoin. For instance, more hydrophobic alcohols such as 1-hexanol and 1-octanol induce faster conversion compared to the less hydrophobic 1-pentanol.^29^ Olafson et al.^68^ demonstrated that hematin crystallization from an organic solvent, such as *n*-octanol, follows a classical layered growth mechanism. In this process, surface diffusion is the primary pathway for molecule incorporation at step edges, exhibiting first-order kinetics and a pronounced asymmetry in molecular attachment from adjacent terraces. As an organic solvent, *n*-octanol plays a crucial role in the crystallization process. They proposed that *n*-octanol molecules form an ordered structure around hematin molecules and at the crystal-solution interface. The subsequent release of these structured solvent molecules may contribute to an entropy increase, facilitating hematin incorporation into growth sites. Vekilov et al.^48^ reported that a lipid-based medium, such as octanol saturated with citrate buffer (CBSO), provides a significantly more favorable environment for hematin crystallization compared to an aqueous medium. The solubility of hematin in CBSO is approximately 100,000 times higher than in aqueous solutions, suggesting that hematin crystallization in the malaria parasite likely occurs within the lipid subphases of the digestive vacuole. Vekilov et al.^69^ based on robust experimental evidence, proposed that β-hematin crystal growth follows a classical mechanism, wherein new crystal layers form via 2D nucleation and grow through solute molecule attachment. Several studies^6,28,29,66,68,70^ suggest that β-hematin formation follows a nucleation and growth process, where small clusters initially form until a critical seed size is reached, after which crystal growth proceeds. Pasternack et al.^29^ proposed a kinetic equation combining elements of first-order chemical and Avrami kinetics, incorporating both monomer hemin concentration and random nucleation/crystal growth. In contrast, Herrera et al.^6^ introduced a model combining second-order and logistic kinetics, considering hematin dimers (instead of monomers) as well as nucleation and growth processes. The discussion above suggests that crystallization in the aqueous-oily medium occurs more rapidly than in the aqueous-acetate medium. This difference in formation mechanisms significantly influences the physicochemical properties of the final products. For example, the smaller particle sizes and broader XRD peaks observed in β-hematin formed in the aqueous-oily medium can be attributed to faster nucleation and growth. These structural differences also impact the magnetic and surface properties, as discussed earlier.

## Conclusions

The physicochemical properties of two β-hematin samples synthesized on either aqueous-acetate or aqueous-oily were found to vary significantly depending on the synthetic media. The X-ray diffraction (XRD) patterns showed broader Bragg peaks for the samples prepared in the aqueous-oily medium compared to those prepared in the aqueous-acetate medium, suggesting a smaller particle size. Further analysis revealed that β-hematin crystals synthesized in both media exhibited larger unit cell volumes in comparison to those reported in the literature. Additionally, scanning electron microscopy showed that crystals formed in the aqueous-acetate medium had a more elongated shape compared to those obtained in the aqueous-oily medium. Surface analysis with X-ray photoelectron spectroscopy indicated that β-hematin crystals synthesized in the aqueous-acetate medium predominantly contained high-spin ferric ions and a high content of hydroxyl groups (OH^–^). In contrast, the surfaces of the crystals synthesized in the aqueous-oily medium contained low- and high-spin ferrous ions, high-spin ferric ions and a lower OH^–^ content. Magnetization versus magnetic applied field curves showed that both paramagnetic and superparamagnetic behaviors at 300 K on the one hand and paramagnetic and ferromagnetic behaviors at 80 K are possible depending on the synthetic media. Moreover, at very low temperatures, antiferromagnetism is predicted, highlighting the complex magnetic properties of β-hematin. Mössbauer spectroscopy revealed distinct differences in the spectral shapes between the two types of β-hematin crystals. The spectra were more asymmetric for the crystals formed in the aqueous-oily medium and less asymmetric for those formed in the aqueous-acetate medium. These findings underscore the significant impact of the synthetic medium on the properties of β-hematin, providing valuable insights into its complex behavior and potential applications.
